# Supplementary material for: Stereochemical Determination of Fistularins Isolated from the Marine Sponge Ecionemia acervus and Their Regulatory Effect on Intestinal Inflammation
Source: Mar Drugs. 2021 Mar 22;19(3):170. doi: 10.3390/md19030170 (PMC8004254; doi:10.3390/md19030170)
Supplement: Supplementary file 1 [file marinedrugs-19-00170-s001.pdf]

Supporting Material

Article

Stereochemical Determination of Fistularins isolated from the Marine Sponge *Ecionemia acervus* and their Regulatory Effect on Intestinal Inflammation

Yeong Kwang Ji[1†], Seon Min Lee[2†], Na-Hyun Kim[2], Nguyen Van Tu[4], Yun Na Kim[3], Jeong Doo Heo[2], Eun Ju Jeong[3*] and Jung-Rae Rho[1*]

[1] Department of Oceanography, Kunsan National University, Gunsan 54150, Republic of Korea; kwang7089@kunsan.ac.kr

[2] Gyeongnam Department of Environment & Toxicology, Korea Institute of Toxicology, 17 Jegok-gil, Munsan-eup 52834, Republic of Korea smlee84@kitox.re.kr(S.M.L.); nhkim@kitox.re.kr (N.-H.K.); jdher@kitox.re.kr(J.D.H.)

[3] Department of Plant & Biomaterials Sciences, Gyeongsang National University, Jinju 52725, Republic of Korea; ; yunna@gntech.ac.kr

[4] Institute of Tropical Biology, 85 Tran Quoc Toan Street District 3, Ho Chi Minh 700000, Vietnam; nguyen.tu@itb.ac.vn

* Correspondence: jrrho@kunsan.ac.kr(J.-R. R.); ejjeong@gntech.ac.kr(E.J.J.); Tel.: +82 63 469 4606(J.-R.R.); +82 55 751 3224 (E.J.J.)

† These authors contributed equally to this work

Contents

Figure S1. CD spectra for compounds **1–6**. --3

Figure S2. ^{1}H and ^{13}C NMR spectra for **1** in CD_3OD. -- 3

Figure S3. ^{1}H and ^{13}C NMR spectra for **2** in CD_3OD ---4

Figure S4. ^{1}H and ^{13}C NMR spectra for **3** in CD_3OD ---5

Figure S5. ^{1}H and ^{13}C NMR spectra for **4** in CD_3OD ---6

Figure S6. ^{1}H and ^{13}C NMR spectra for **5** in CD_3OD ---7

Figure S7. ^{1}H and ^{13}C NMR spectra for **6** in CD_3OD ---8

Figure S8. (A) ^{1}H NMR and (B) ^{13}C NMR spectra of **3** in acetone-d_6. -------------------------9

Figure S9. ^{1}H NMR spectra for (A) (*S*)-MTPA ester

and (B) (*R*)-MTPA ester of **3** in $CDCl_3$. ---10

Figure S10. COSY NMR spectra for (A) (*S*)-MTPA ester

and (B) (*R*)-MTPA ester of **3** in $CDCl_3$.--11

Table S1. NMR Chemical shifts for compounds **1-2** (500MHz for ^{1}H, in CD_3OD).-----------12

Table S2. Calculated Carbon Shielding Tensors for Conformers of **1a**-------------------------14

Table S3. Calculated Proton Shielding Tensors for Conformers of **1a** -------------------------15

Table S4. Calculated Carbon Shielding Tensors for Conformers of **1b** ------------------------16

Table S5. Calculated Proton Shielding Tensors for Conformers of **1b** -------------------------17

Table S6. Calculation of DP4+ probability for **1a** and **1b.** -------------------------------------17

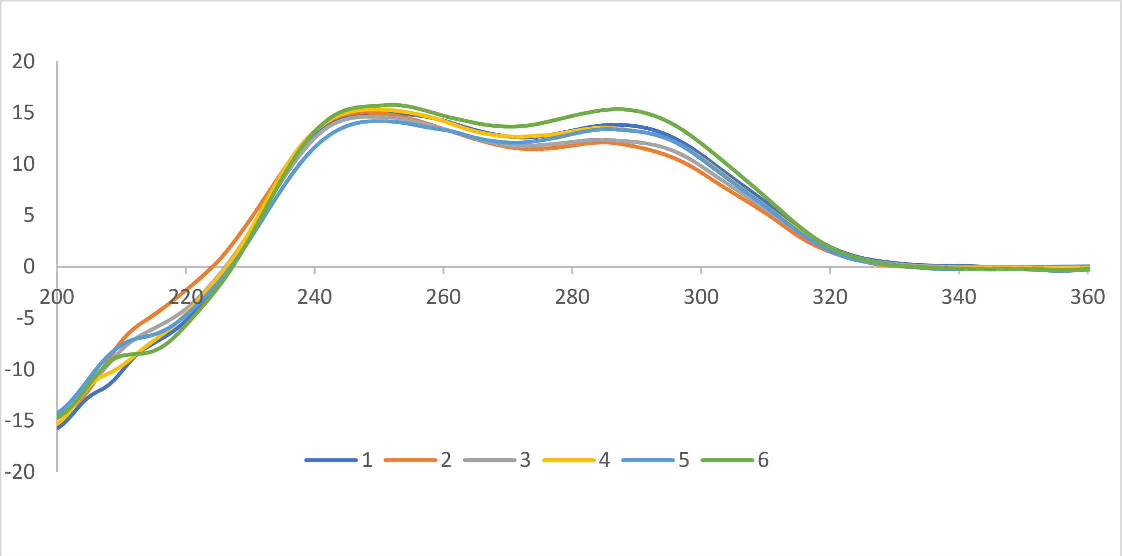

Figure S1. CD spectra for compounds **1−6**.

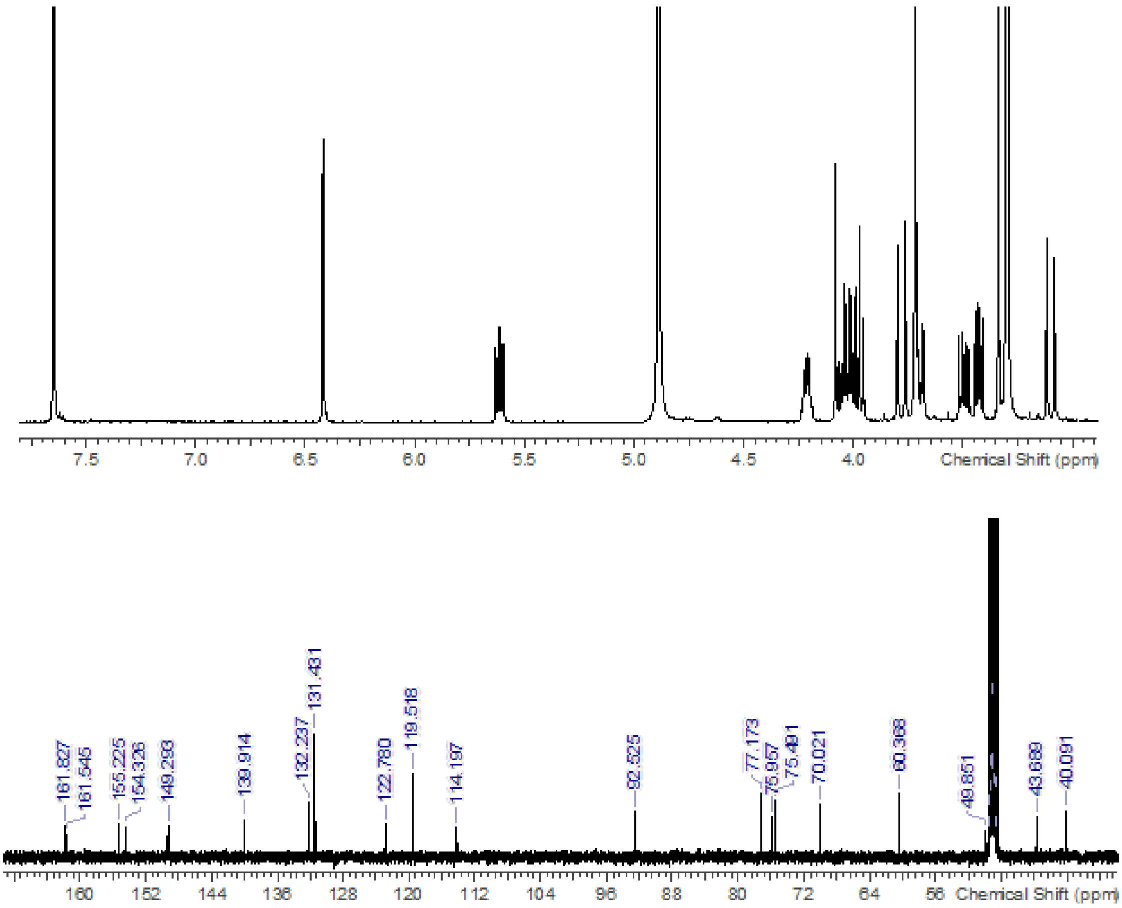

Figure S2. ^{1}H and ^{13}C NMR spectra for **1** in CD_3OD.

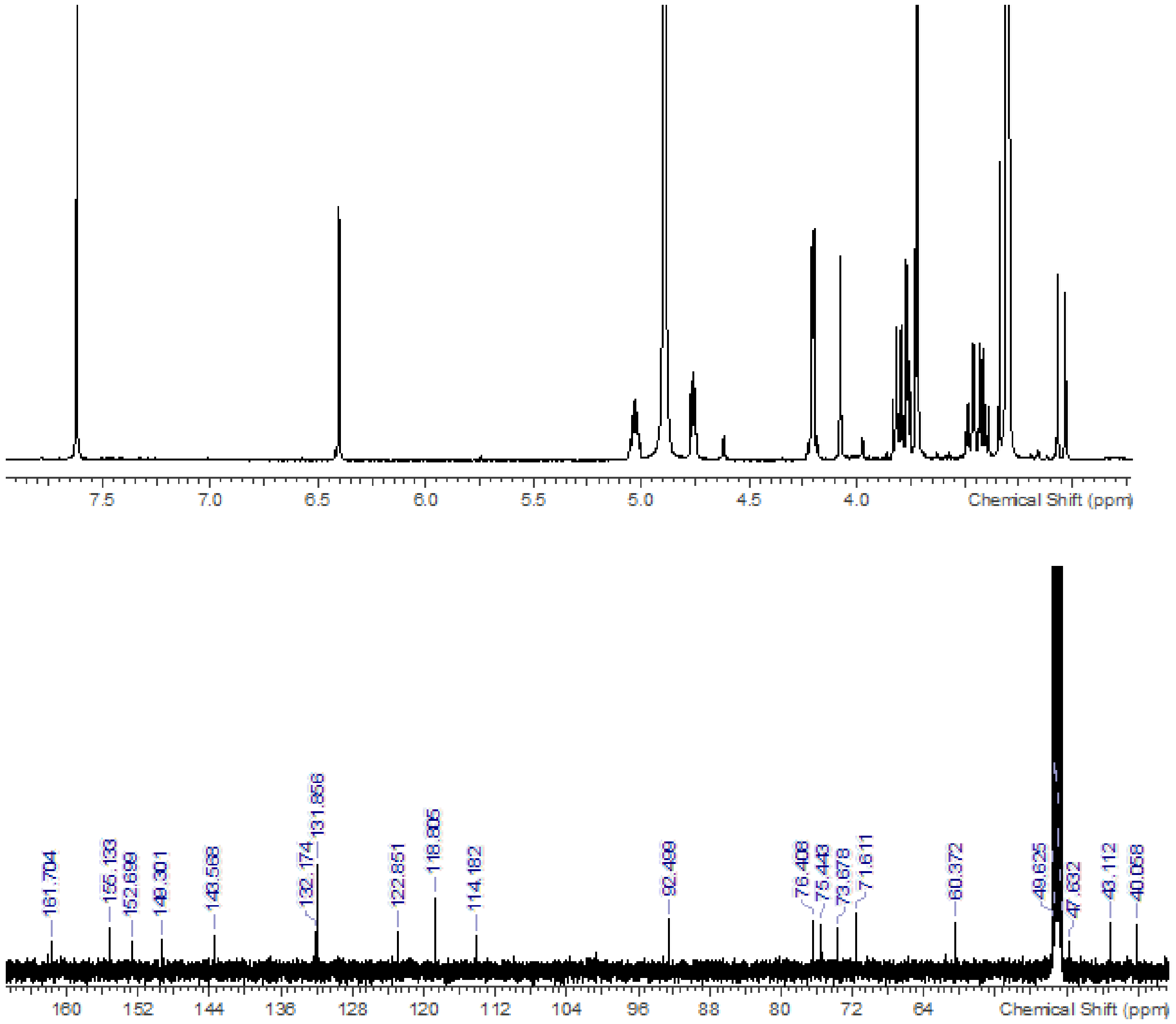

Figure S3. ^{1}H and ^{13}C NMR spectra for **2** in CD_3OD.

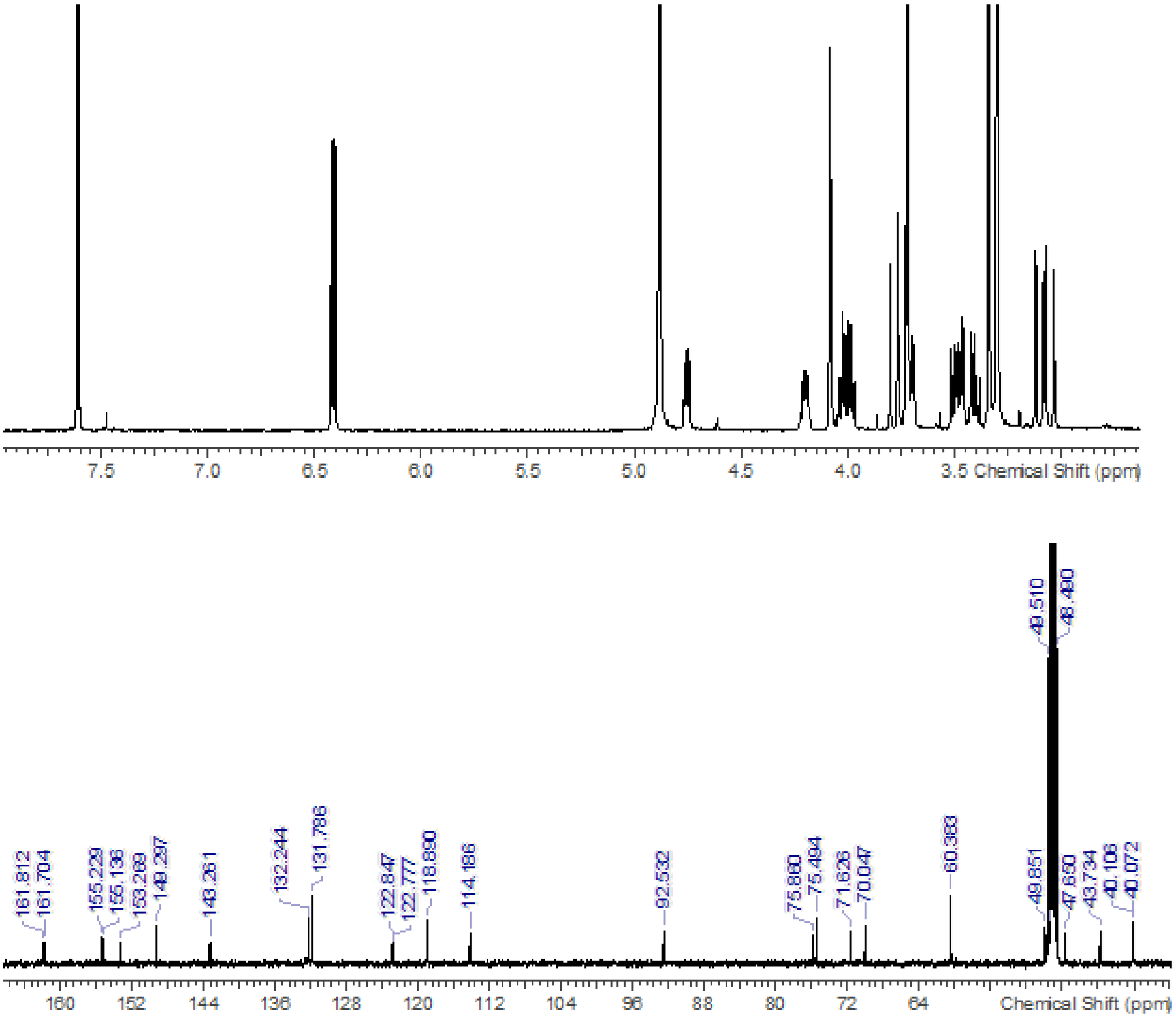

Figure S4. ^{1}H and ^{13}C NMR spectra for **3** in CD_3OD.

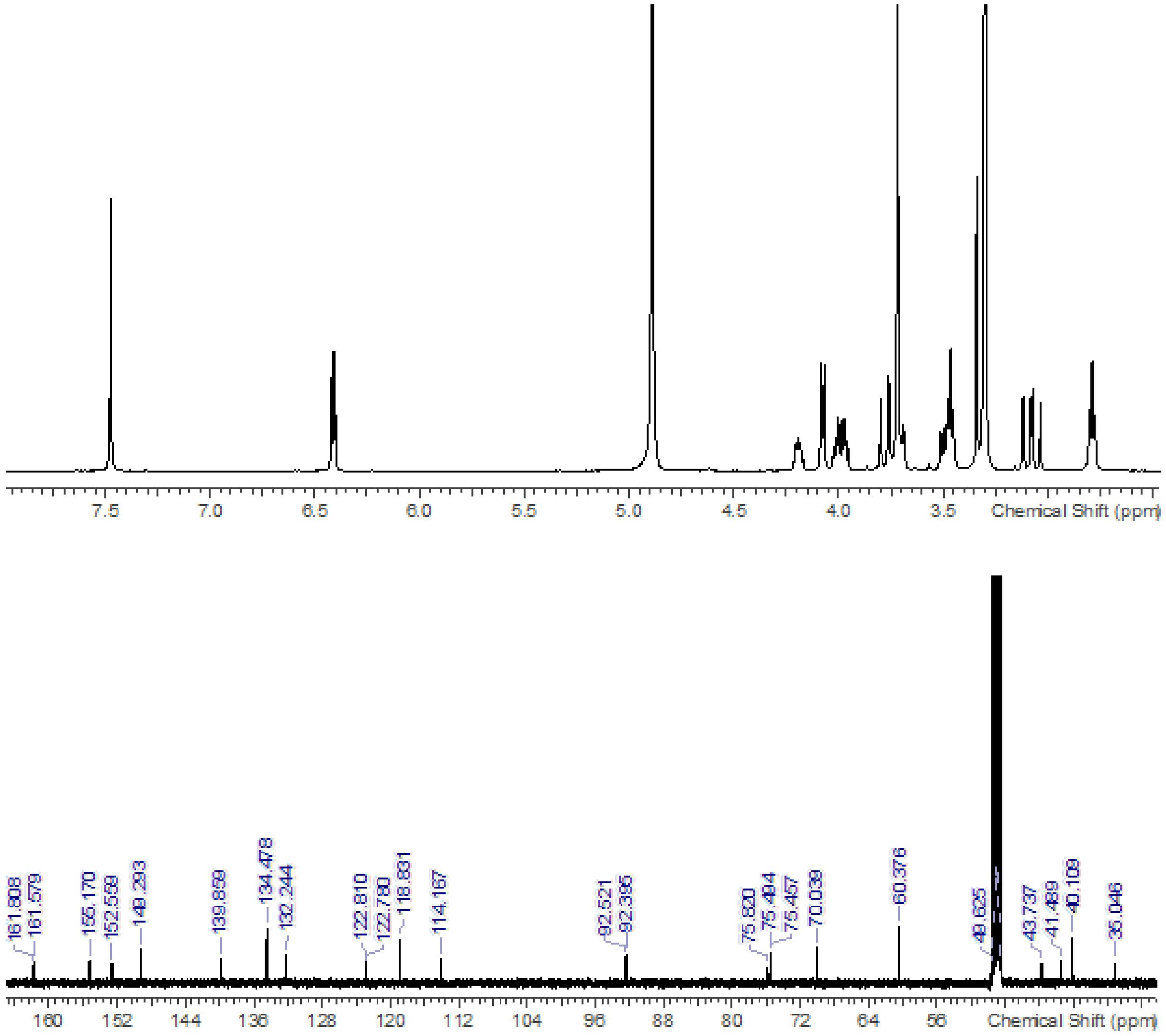

Figure S5. ^{1}H and ^{13}C NMR spectra for **4** in CD_3OD.

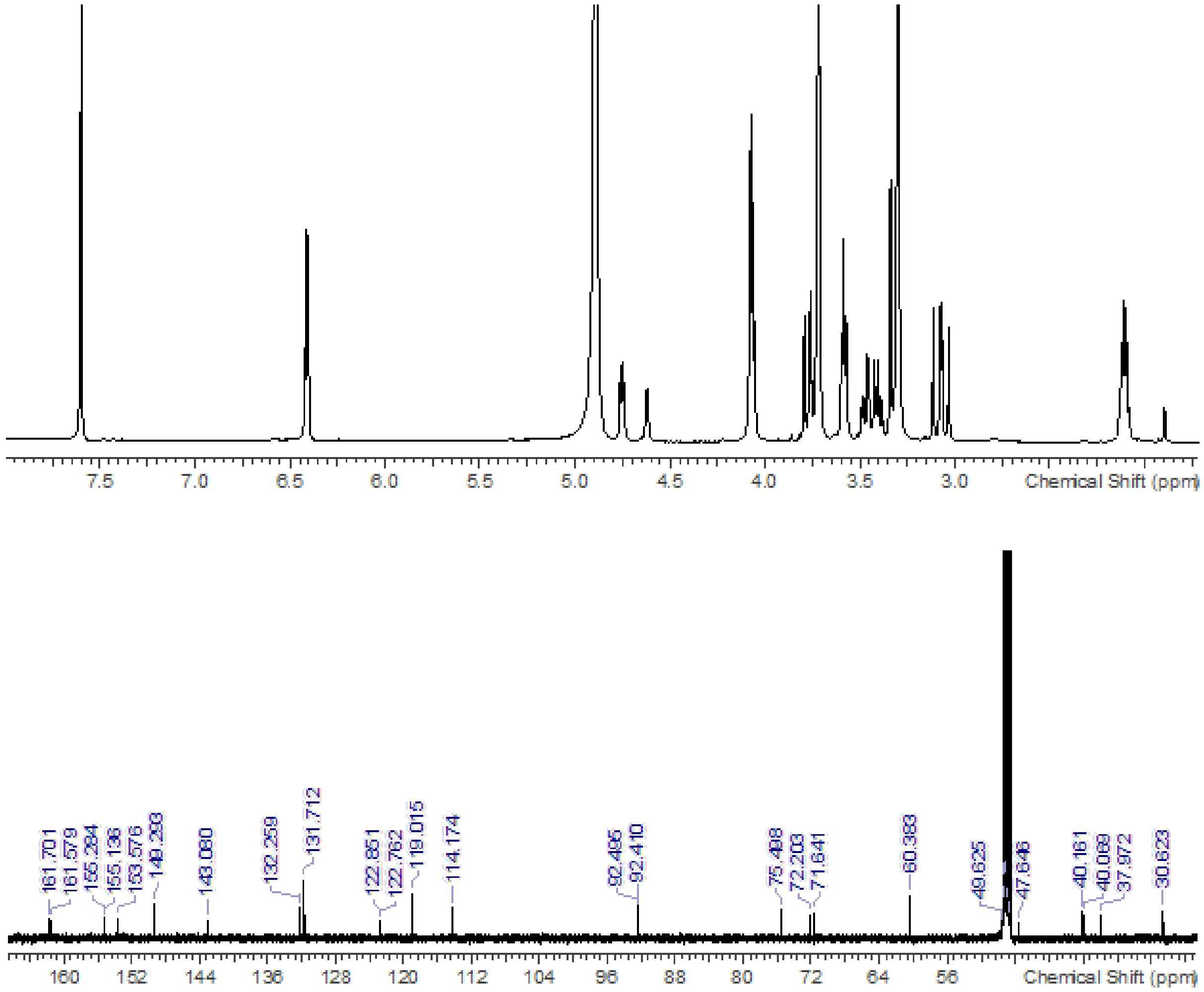

Figure S6. ^{1}H and ^{13}C NMR spectra for **5** in CD_3OD.

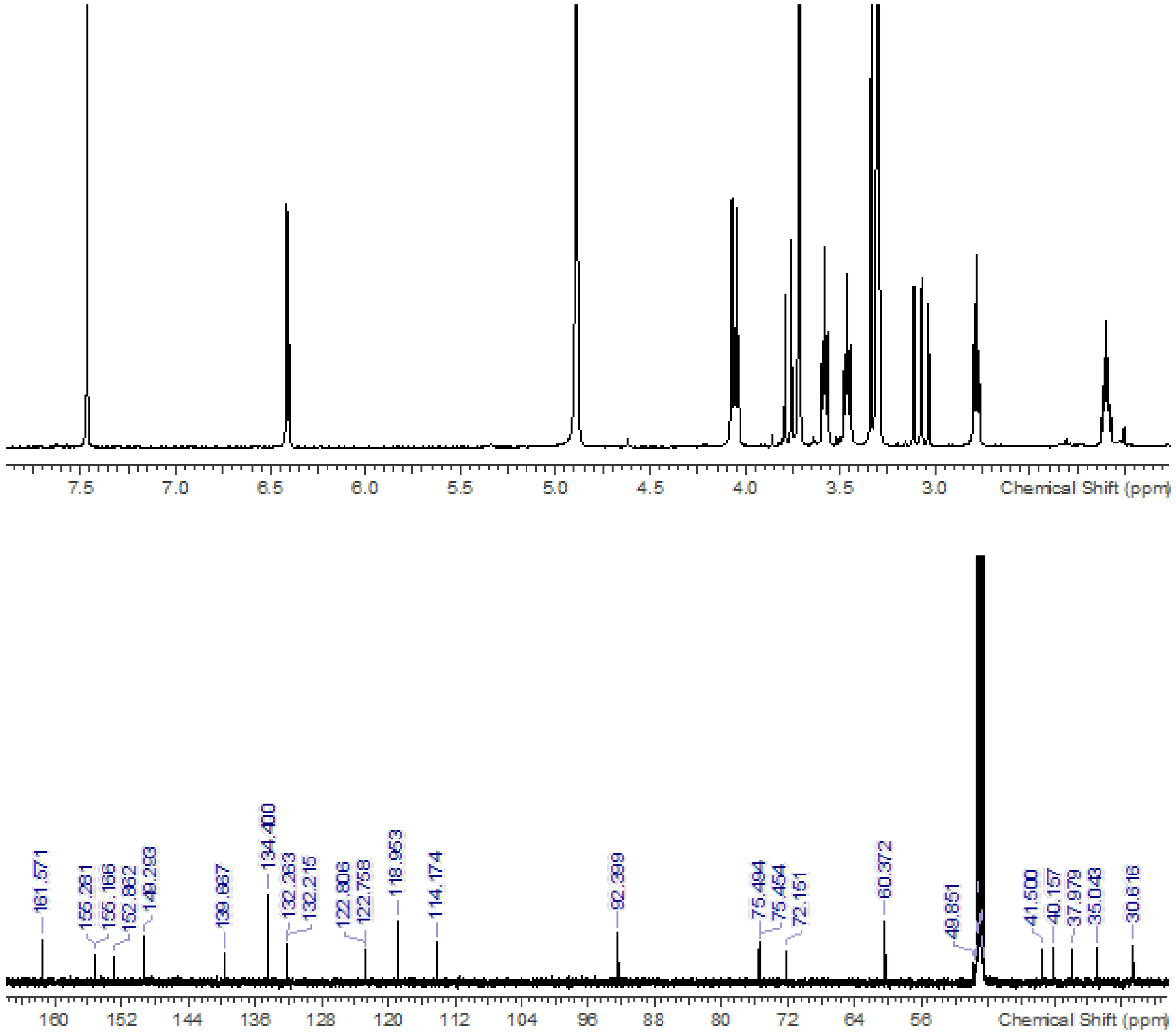

Figure S7. ^{1}H and ^{13}C NMR spectra for **6** in CD_3OD.

(A)

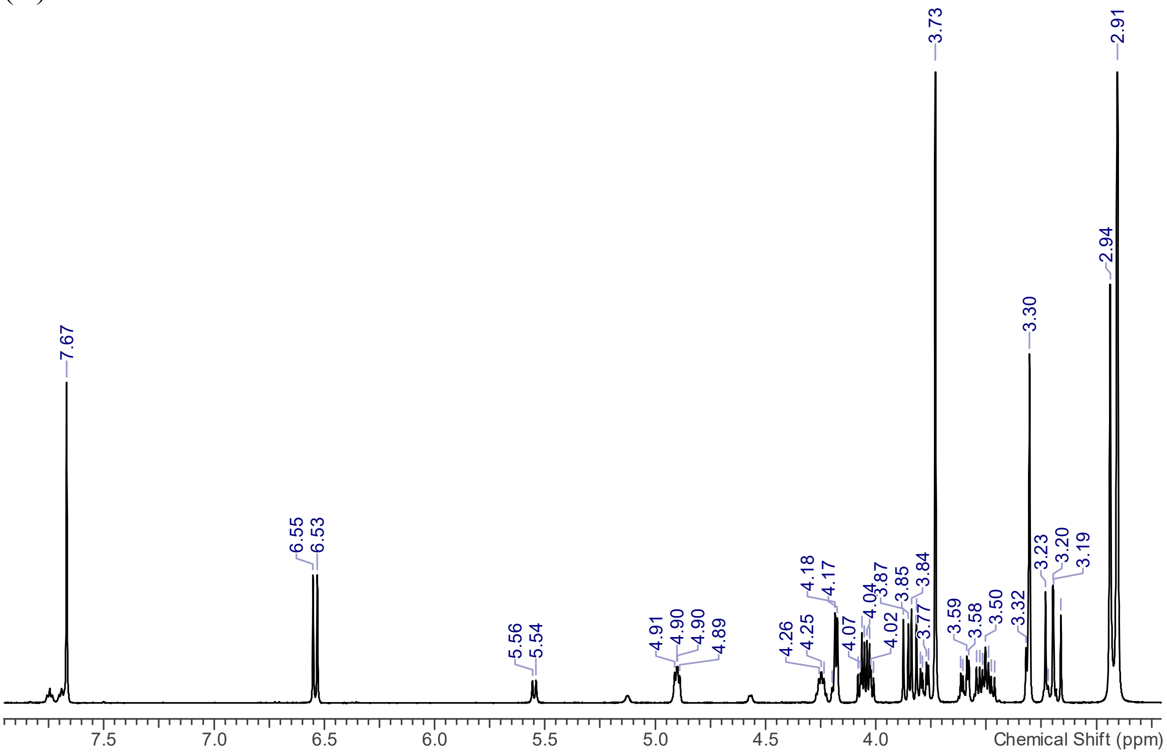

(B)

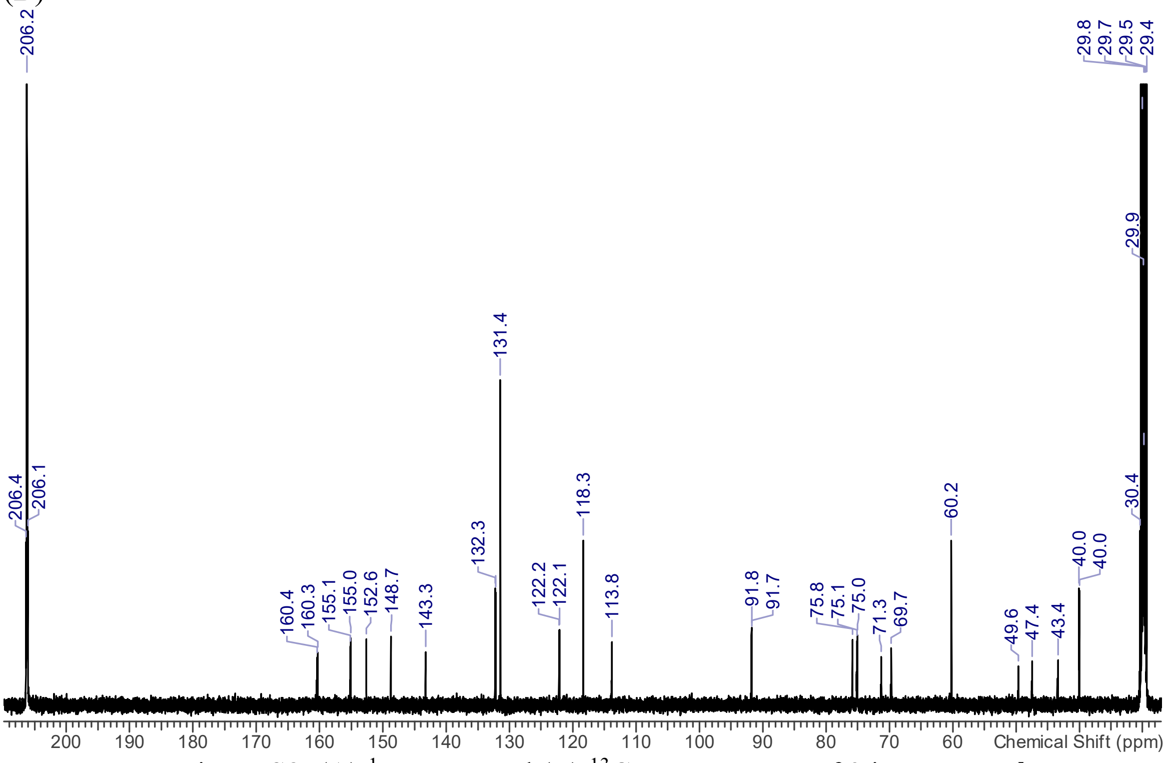

Figure S8. (A) ^{1}H NMR and (B) ^{13}C NMR spectra of **3** in acetone-d_6.

(A)

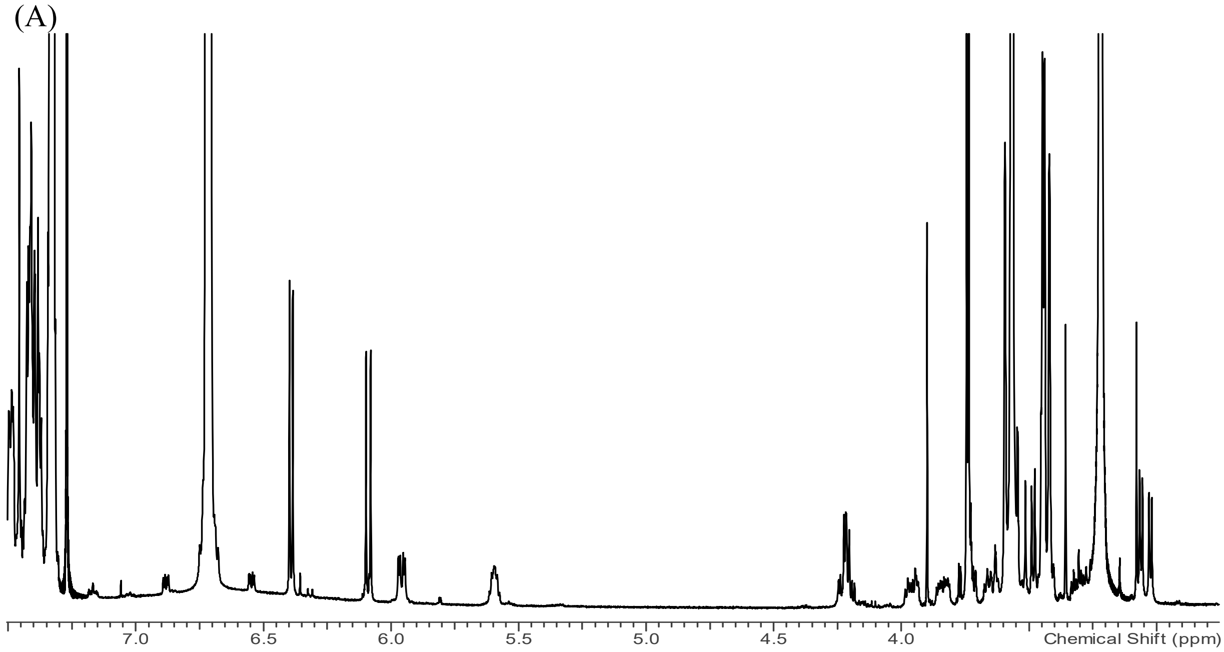

(B)

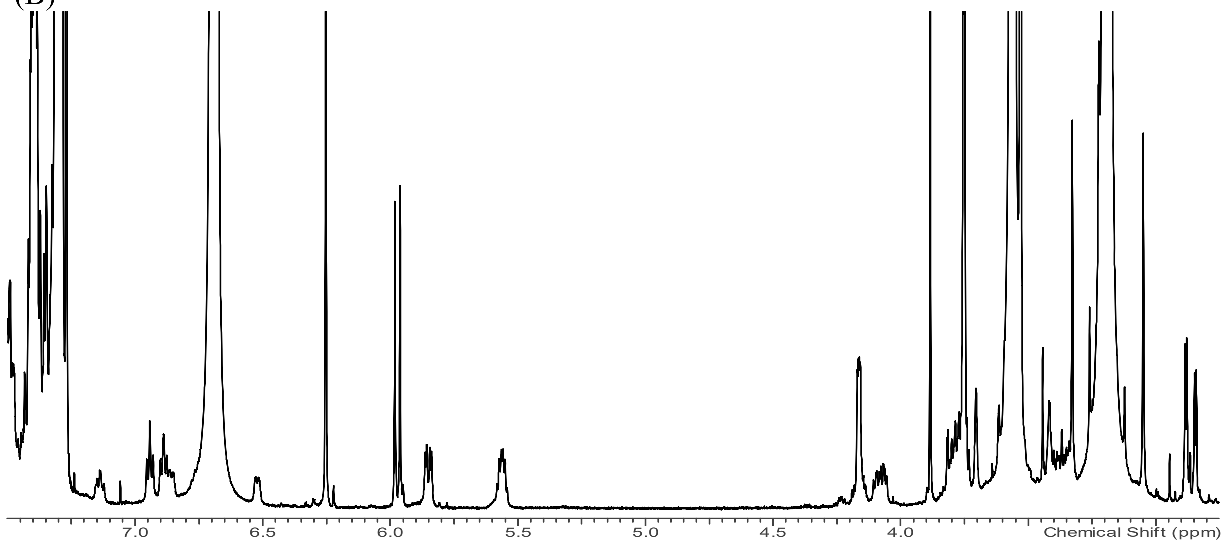

Figure S9. ^{1}H NMR spectra for (A) (*S*)-MTPA ester and (B) (*R*)-MTPA ester of **3** in $CDCl_3$.

(A)

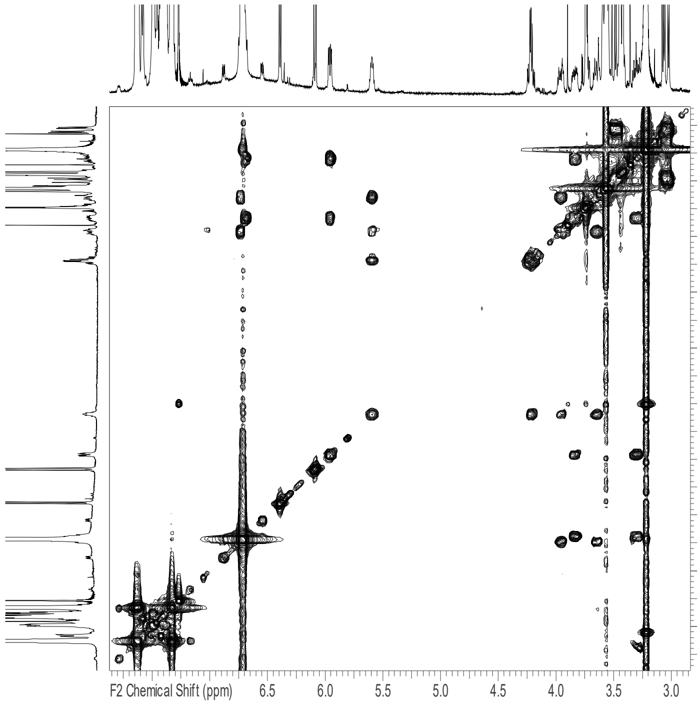

(B)

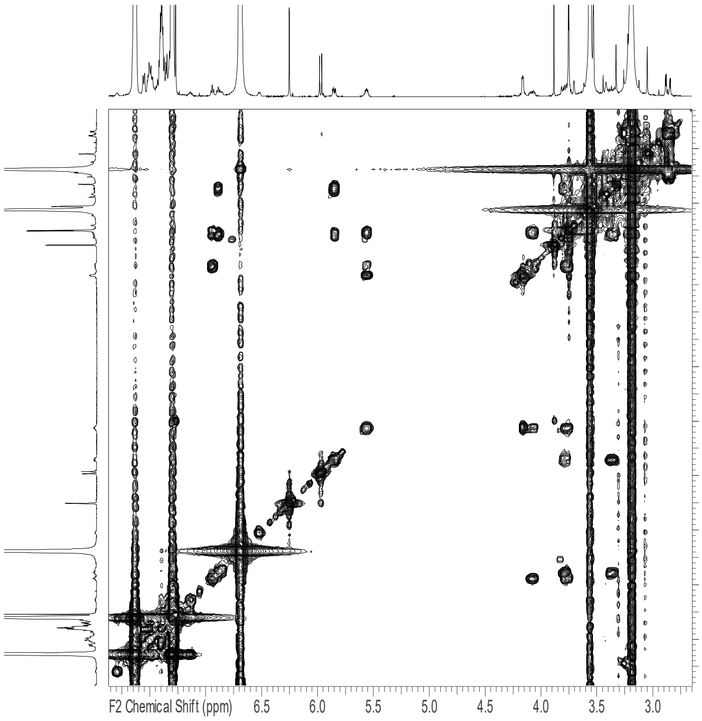

Figure S10. COSY NMR spectra for (A) (*S*)-MTPA ester and (B) (*R*)-MTPA ester of **3** in $CDCl_3$.

Table S1. NMR Chemical shifts for compounds **1-2** (500MHz for ^{1}H, in CD_3OD)

	**FS-1 (1)**		**FS-2 (2)**	
	1H	13C	1H	13C
1, 1'	4.08, s	75.5, CH	4.08, s	75.4, CH
2, 2'		114.2, C		114.2, C
3, 3'		149.3, C		149.3, C
4, 4'		122.8, C		122.9, C
5, 5'	6.43, s	132.2, CH	6.41, s	132.2, CH
6, 6'		92.5, C		92.5, C
7, 7'	3.10, d (18.3)	40.1, CH_2	3.05, d (18.4)	40.1, CH_2
	3.78, d (18.3)		3.74, d (18.4)	
8, 8'		155.3, C		155.1, C
9, 9'		161.8, C		161.7, C
10	3.50, dd (13.7, 7.7)	43.7, CH_2	3.77, t (8.8)	43.1, CH_2
	3.70, dd (13.7, 4.7)		3.82, dd (8.8, 6.4)	
11	4.22, m	70.0, CH	5.03, m	76.4, CH
12	4.01, dd (9.3, 5.4)	76.0, CH_2	4.19, dd (10.5, 3.9)	73.7, CH_2
	4.05, dd (9.3, 5.4)		4.05, dd (10.5, 4.4)	
13		154.3, C		152.7, C
14, 14'		119.5, C		118.8, C
15, 15'	7.65, s	131.4, CH	7.61, s	131.9, CH
16		139.9, C		143.3, C
17	5.61, dd (9.1, 7.1)	77.2, CH	4.76, dd (7.3, 4.6)	71.6, CH
18	3.43, dd (9.1, 7.1)	49.1, CH_2	3.41, dd (13.7, 7.3)	47.6, CH_2
	3.97, t (9.1)		3.47, dd (13.7, 4.9)	
OCH_3	3.72, s	60.4, CH_3	3.72, s	60.4, CH_3

Table S1. ^{1}H NMR Chemical shifts for compounds **3-6** (500 MHz, in CD3OD)

no	**FS-3 (3)**	**17-deoxyFS-3 (4)**	**11-deoxyFS-3 (5)**	**11,17-dideoxyFS-3 (6)**
1, 1'	4.08, s	4.07, s / 4.08, s	4.08, s	4.07, s
5, 5'	6.41, s / 6.42, s	6.41, s / 6.42, s	6.41, s / 6.42, s	6.41, s / 6.42, s
7, 7'	3.05 / 3.75, d	3.05 / 3.74, d	3.05 / 3.74, d	3.05 / 3.74, d (18.1)
	(18.3) 3.10 / 3.78,	(18.3) 3.10 / 3.78,	(18.3) 3.10 / 3.78,	3.09 / 3.77, d (18.1)
10	d (18.3)	d (18.3)	d (18.3)	3.58, t (6.9)
	3.50, dd (13.9, 7.6)	3.50, dd (13.9, 7.6)	3.58, t (7.1)	3.58, t (6.9)
11	3.71, dd (13.9, 4.7)	3.71, dd (13.9, 4.4)	3.58, t (7.1)	2.10, m
12	4.20, m	4.19, m	2.11, m	4.05, t (6.1)
	3.98, dd (9.1, 5.6)	3.98, dd (9.1, 5.4)	4.06, t (6.1)	4.05, t (6.1)
15,	4.03, dd (9.1, 5.6)	4.01, dd, 9.1, 5.4)	4.06, t (6.1)	7.47, s
15'	7.60, s	7.48, s	7.60, s	2.78, t (7.3)
17	4.76, dd (7.3, 4.7)	2.79, t (7.1)	4.75, dd (7.3, 4.9)	3.46, t (7.3)
18	3.41, dd (13.7, 7.3)	3.47, t (7.1)	3.40, dd (13.7, 7.3)	3.46, t (7.3)
	3.47, dd (13.7, 4.7)	3.47, t (7.1)	3.47, dd (13.7, 4.9)	3.72, s
OCH_3	3.72, s	3.72, s	3.72, s	

Table S1. ^{13}C NMR Chemical shifts for compounds **3-6** (125 MHz, in CD_3OD)

no	**FS-3 (3)**	**17-deoxyFS-3 (4)**	**11-deoxyFS-3 (5)**	**11,17-dideoxyFS-3 (6)**
1, 1’	75.5 / 75.5, CH	75.5 / 75.5, CH	75.4 / 75.5, CH	75.5 / 75.5, CH
2, 2’	114.2 / 114.2, C	114.2 / 114.2, C	114.2 / 114.2, C	114.2 / 114.2, C
3, 3’	149.3 / 149.3, C	149.3 / 149.3, C	149.3 / 149.3, C	149.3 / 149.3, C
4, 4’	122.8 / 122.9, C	122.8 / 122.8, C	122.8 / 122.9, C	122.8 / 122.9, C
5, 5’	132.2 / 132.3,	132.2 / 132.2, CH	132.2 / 132.3, CH	132.2 / 132.3, CH
6, 6’	CH	92.4 / 92.5, C	92.4 / 92.5, C	92.4 / 92.4, C
7, 7’	92.5 / 92.5, C	40.1 / 40.1, CH_2	40.1 / 40.2, CH_2	40.1 / 40.2, CH_2
8, 8’	40.1 / 40.1, CH_2	155.2 / 155.2, C	155.1 / 155.2, C	155.2 / 155.3, C
9, 9’	155.1 / 155.2, C	161.6 / 161.8, C	161.6 / 161.7, C	161.6 / 161.6, C
10	161.7 / 161.8, C	43.7, CH_2	38.0, CH_2	38.0, CH_2
11	43.7, CH_2	70.0, CH	30.6, CH	30.6, CH
12	70.0, CH	75.8, CH_2	72.2, CH_2	72.2, CH_2
13	75.9, CH_2	152.6, C	153.6, C	152.9, C
14, 14’	153.3, C	118.8 / 118.8, C	119.0 / 119.0, C	119.0 / 119.0, C
15, 15’	118.9 / 118.9, C	134.5 / 134.5, CH	131.7 / 131.7, CH	134.4 / 134.4, CH
16	143.3 / 143.3, CH	139.9, C	143.1, C	139.7, C
17	143.3, C	35.0, CH	71.6, CH	35.0, CH
18	71.6, CH	41.5, CH_2	47.6, CH_2	41.5, CH_2
OCH_3	47.6, CH_2	60.4, CH_3	60.4, CH_3	60.4, CH_3
	60.4, CH_3			

*Consistent chemical shifts were highlighted with the same color.

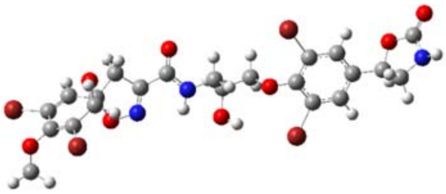

1a (17*R*)

Table S2. Calculated Carbon Shielding Tensors for Conformers of **1a**

conformers	**1**	**2**	**3**	**4**	**5**	**6**
Boltzmann Distribution	0.32	0.27	0.15	0.09	0.09	0.08
1	109.7	109.4	110.6	110.1	109.7	109.9
2	47.9	48.3	46.0	47.4	46.9	47.5
3	29.7	29.8	29.9	30.0	29.8	30.3
4	40.1	40.6	39.9	39.7	39.9	40.5
5	47.9	47.6	47.2	48.2	47.7	47.5
6	91.6	91.7	91.2	92.5	92.7	92.9
7	145.0	144.8	145.0	144.7	144.5	144.5
8	25.6	25.7	24.7	24.1	24.1	24.6
9	17.8	17.9	20.1	23.6	23.6	23.7
10	140.4	140.1	146.3	144.1	143.9	144.1
11	114.1	114.2	115.1	111.9	112.3	111.9
12	113.9	113.9	109.4	110.3	110.2	110.3
13	28.3	28.4	24.7	28.3	28.3	28.4
14	45.8	44.5	44.6	44.9	45.7	44.4
14	44.4	45.8	45.5	45.9	44.6	45.8
15	51.8	53.4	46.5	51.2	51.2	52.3
15	53.8	51.8	49.7	50.8	51.8	51.5
16	41.1	41.0	43.2	43.6	43.8	43.6
17	109.6	109.7	106.6	107.1	107.2	107.6
18	135.2	134.9	138.5	133.8	134.1	134.4
9′	21.6	22.0	21.6	21.6	21.7	21.8
OCH_3	126.4	126.3	127.4	126.5	126.4	126.6

Table S3. Calculated Proton Shielding Tensors for Conformers of **1a**

conformers	**1**	**2**	**3**	**4**	**5**	**6**
Boltzmann Distribution	0.32	0.27	0.15	0.09	0.09	0.08
1	27.58	27.53	27.94	27.66	27.60	27.62
5	25.38	25.40	25.42	25.35	25.30	25.39
7	28.63	28.58	28.82	27.60	27.52	27.61
7	27.64	27.68	28.04	28.67	28.65	28.64
10	28.08	28.07	27.80	28.33	28.27	28.31
10	27.47	27.51	27.37	27.30	27.40	27.35
11	28.03	28.02	29.01	28.02	28.00	28.01
12	27.81	27.82	27.30	27.20	27.21	27.35
12	27.09	27.02	28.60	27.79	27.70	27.62
15	24.15	23.97	24.03	23.72	24.15	23.68
15	24.04	24.14	23.58	24.16	23.75	24.11
17	26.11	26.12	26.36	26.26	26.19	26.18
18	28.46	28.38	27.89	28.54	28.63	28.62
18	27.64	27.59	27.59	27.99	28.01	27.99
OCH_3	27.86	27.87	27.98	28.23	28.23	28.26
OCH_3	27.88	27.88	27.92	27.88	27.87	27.88
OCH_3	28.25	28.20	28.19	27.92	27.90	27.91

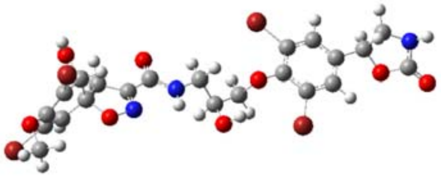

1b (17*S*)

Table S4. Calculated Carbon Shielding Tensors for Conformers of **1b**

conformers	**1**	**2**	**3**	**4**	**5**	**6**
Boltzmann Distribution	0.18	0.18	0.16	0.16	0.16	0.15
1	110.1	110.0	109.5	110.2	110.0	110.4
2	47.3	47.6	47.8	47.4	47.6	47.7
3	30.5	29.9	30.3	30.0	30.2	29.8
4	40.5	40.0	40.0	40.5	40.4	39.6
5	47.9	47.5	47.4	48.0	47.8	48.0
6	92.7	92.7	92.6	92.6	92.7	92.6
7	144.5	144.6	144.4	144.5	144.4	144.8
8	24.1	24.2	24.4	23.8	23.9	23.7
9	23.8	23.9	23.6	23.7	23.8	24.0
10	143.7	143.7	143.5	143.3	143.4	143.5
11	111.9	112.0	112.1	112.4	112.5	112.1
12	110.1	110.1	110.1	110.4	110.2	110.2
13	28.4	28.3	28.9	28.5	28.6	28.2
14	44.9	45.6	44.6	45.5	45.1	44.9
14	45.5	44.9	45.3	45.2	45.7	45.5
15	51.7	50.5	52.5	51.1	52.1	51.5
15	50.8	51.4	51.6	52.1	51.2	50.7
16	43.7	43.5	43.5	43.7	43.5	43.6
17	107.0	106.9	107.5	107.3	107.5	106.9
18	134.0	133.9	134.4	134.1	134.2	133.7
9′	21.7	21.7	21.9	21.8	21.7	21.7
OCH_3	126.5	126.4	126.3	126.4	126.4	126.5

Table S5. Calculated Proton Shielding Tensors for Conformers of **1a**

conformers	**1**	**2**	**3**	**4**	**5**	**6**
Boltzmann Distribution	0.18	0.18	0.16	0.16	0.16	0.15
1	25.36	25.37	25.37	25.36	25.38	25.29
5	27.64	27.60	27.62	27.65	27.66	27.57
7	27.63	27.51	27.58	27.60	27.59	27.57
7	28.72	28.67	28.69	28.67	28.67	28.63
10	28.35	28.35	28.29	28.30	28.30	28.31
10	27.39	27.31	27.43	27.38	27.47	27.39
11	28.01	28.03	27.98	27.99	27.96	28.00
12	27.26	27.33	27.18	27.31	27.20	27.18
12	27.76	27.70	27.73	27.66	27.71	27.75
15	24.15	23.72	23.68	23.72	24.14	23.67
15	23.71	24.14	24.13	24.13	23.70	24.16
17	26.23	26.20	26.19	26.26	26.17	26.16
18	28.00	28.02	28.01	27.99	28.01	28.01
18	28.53	28.54	28.60	28.52	28.60	28.61
OCH_3	27.86	27.95	27.93	27.90	27.89	27.83
OCH_3	28.27	28.22	28.21	28.21	28.22	28.23
OCH_3	27.86	27.90	27.89	27.89	27.89	27.87

Table S6. Calculation of DP4+ probability for **1a** and **1b**

Functional	Solvent?	Basis Set	Type of Data
mPW1PW91	PCM	6-311+G(d,p)	Shielding Tensors

	Isomer 1	Isomer 2	Isomer 3	Isomer 4	Isomer 5	Isomer 6
sDP4+ (H data)	100.00%	0.00%	-	-	-	-
sDP4+ (C data)	94.73%	5.27%	-	-	-	-
sDP4+ (all data)	100.00%	0.00%	-	-	-	-
uDP4+ (H data)	99.96%	0.04%	-	-	-	-
uDP4+ (C data)	95.71%	4.29%	-	-	-	-
uDP4+ (all data)	100.00%	0.00%	-	-	-	-
DP4+ (H data)	100.00%	0.00%	-	-	-	-
DP4+ (C data)	99.75%	0.25%	-	-	-	-
DP4+ (all data)	100.00%	0.00%	-	-	-	-

Where, Isomer 1= **1a**, Isomer 2 = **1b**
